# Supplementary figures and images for: Laboratory correlates of SARS-CoV-2 seropositivity in a nationwide sample of patients on dialysis in the U.S
Source: PLoS One. 2021 Apr 15;16(4):e0249466. doi: 10.1371/journal.pone.0249466 (PMC8049224; doi:10.1371/journal.pone.0249466)

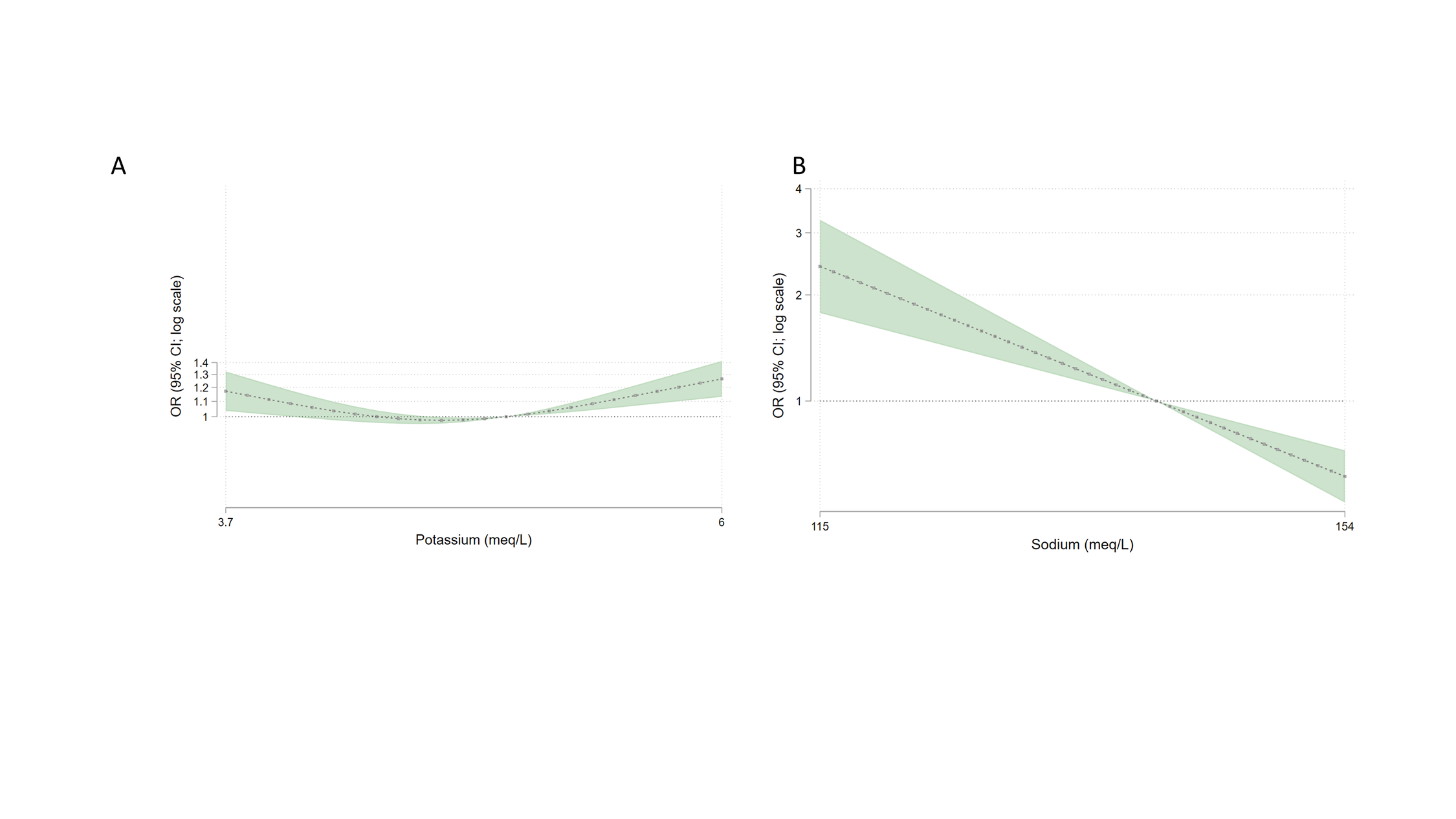

Supplement: S1 Fig — Serum potassium (Panel A) and sodium (Panel B) concentrations < 5 meq/L and < 140 meq/L respectively were associated with higher odds of seropositivity. For potassium, there was also a higher odds for seropositivity at potassium concentrations above 5.0 meq/L. (TIF) [file pone.0249466.s001.tif]

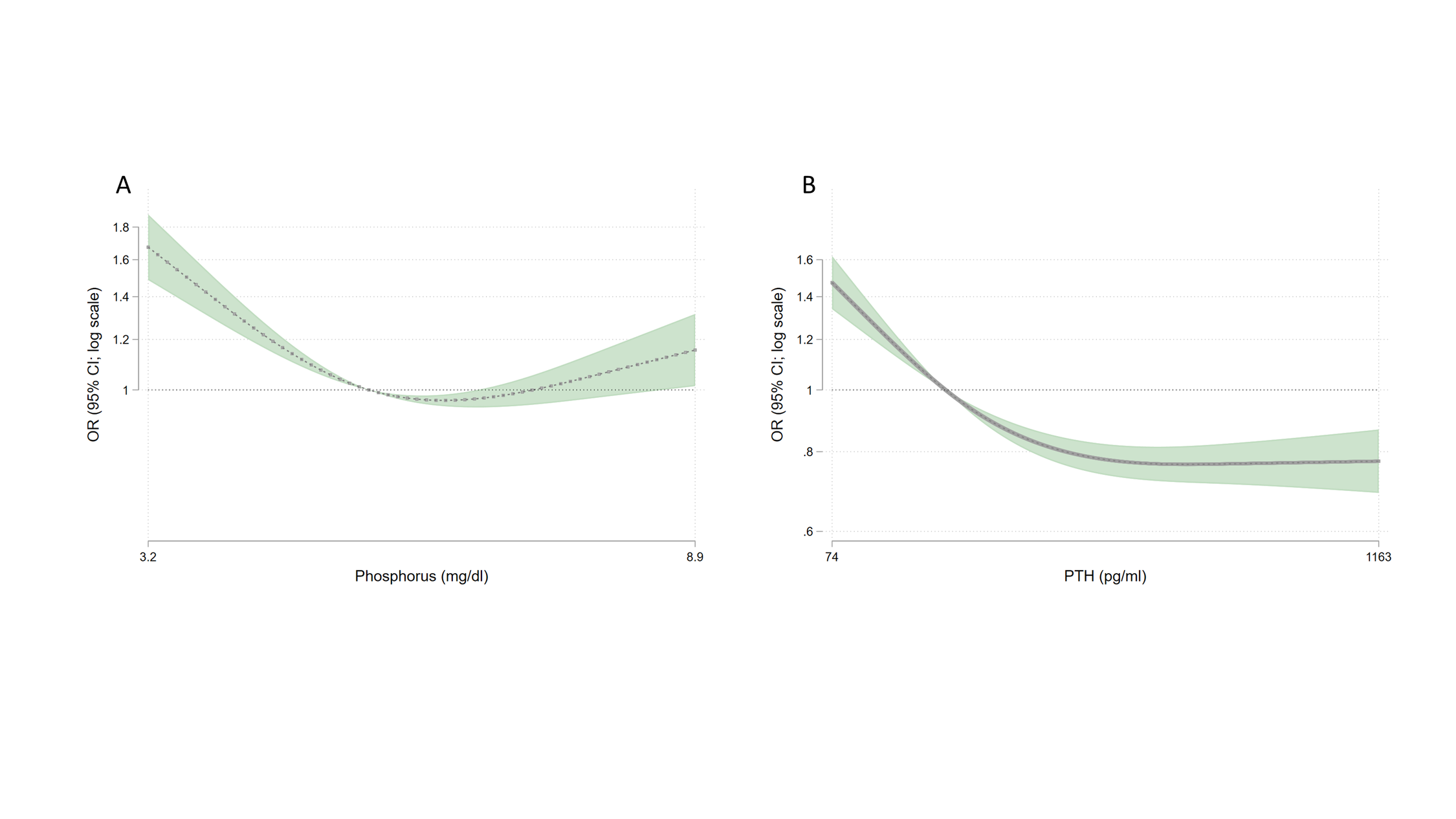

Supplement: S2 Fig — Panels A and B show odds of seropositivity compared with reference concentrations of 5.5 mmol/L and 300 pg/mL for phosphate and PTH respectively. (TIF) [file pone.0249466.s002.tif]

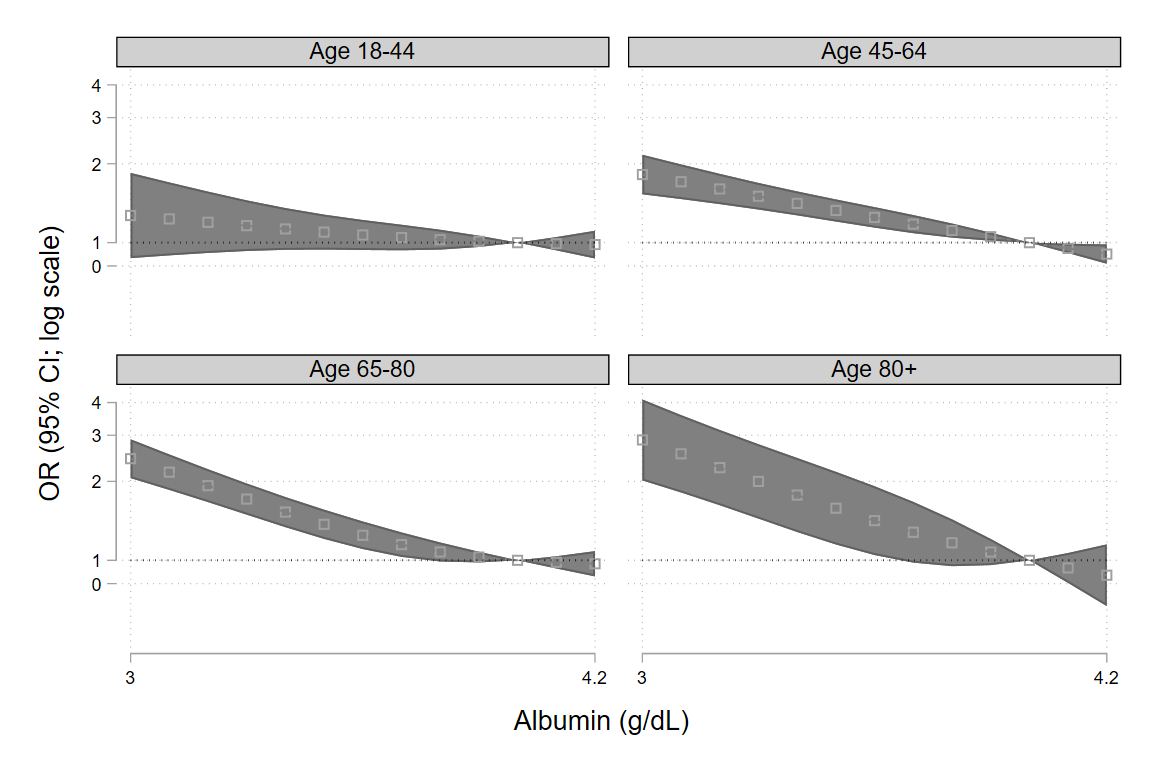

Supplement: S3 Fig — In evaluating whether age modified the association between albumin and seropositivity, we found that the overall relationship was similar across age categories. Older patients however had higher odds of SARS-CoV-2 seropositivity than younger patients at serum albumin concentrations below 4 g/dL vs. a concentration at 4 g/dL (p value for interaction = 0.0035). (TIF) [file pone.0249466.s003.tif]

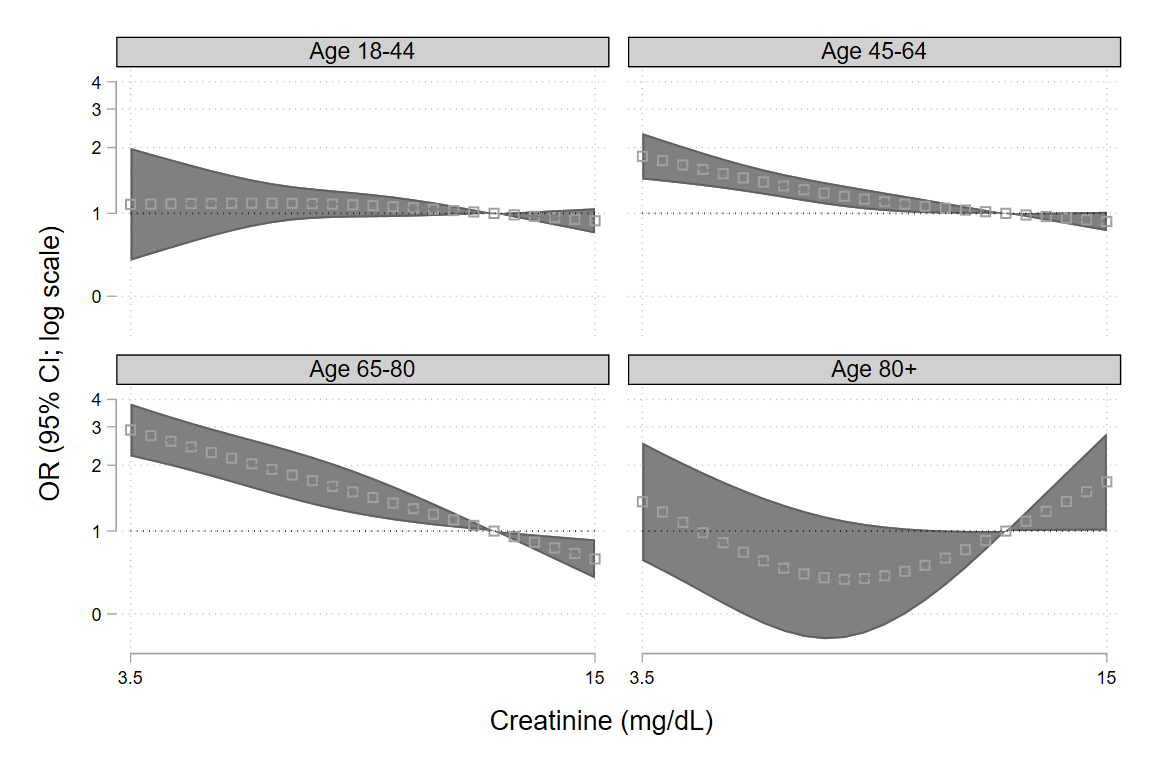

Supplement: S4 Fig — For serum creatinine, a similar trend holds true, i.e., that overall patients with lower serum creatinine had higher odds for seropositivity, and that older patients had higher odds of SARS-CoV-2 seropositivity than younger patients at serum creatinine concentration 12.5 mg/dL (p value for interaction = 0.0058). However among the 80 years or above category, a small number of persons had serum creatinine concentration 12.5 mg/dL or higher, thereby obscuring this trend. (TIF) [file pone.0249466.s004.tif]
